# Employing quality improvement methodology in sepsis: an electronic sepsis order set further improves compliance with the Surviving Sepsis Campaign 3-hour bundle

**DOI:** 10.1186/cc13553

**Published:** 2014-03-17

**Authors:** S Rossi, A Shields, N Gauge, M Kinirons, A Hopper, G Glover, R Beale

**Affiliations:** 1 Guys and St Thomas NHS Foundation Trust, London, UK

## Introduction

The Surviving Sepsis Campaign (SSC) has developed guidelines to promote evidence-based management for patients with severe sepsis [1]. Improvements in bundle compliance have been demonstrated over time, but compliance remains below 40%. Sepsis has been studied in acute and critical care environments, but little research has focused on the management in level 1 wards. An initial audit of patients with sepsis who were referred to the GSTT critical care outreach team revealed very low overall compliance to the SSC 3-hour bundle. A novel quality improvement campaign was instituted with the aim of improving bundle compliance.

## Methods

A retrospective cohort study in a university hospital was performed. Patients on level 1 wards with severe sepsis registered in the adult critical care response team (CCRT) database in November 2012 were identified (Cohort A). Physiological observation, track and trigger scores, compliance with the 3-hour bundle elements (measured lactate, blood cultures before antibiotics, fluid challenge, early antibiotics), antimicrobial stewardship and 28-day mortality were recorded. Following this, a quality improvement project was initiated: central to this was an electronic 'SEPSIS' order set, containing appropriate investigations and a step-by-step management guide for use on level 1 wards. A 'viral' print and social media campaign were also undertaken. Compliance to the SSC early care bundle was re-examined in two cohorts of patients in July 2013; patients that were referred to the CCRT as before (Cohort B) and also patients who had the electronic order set activated (Cohort C).

## Results

The mean age of all patients studied (*n *= 79) was 66.5 years. Fifty-three per cent of the patients were male. Thirty-one per cent were in septic shock at the time of sepsis identification. Overall SSC bundle compliance was 6.60% (Cohort A), 24% (Cohort B) and 45.5% (Cohort C). Improvements in other bundle parameters were also seen, including blood culture (54%, 72%, 91%), antibiotic administration (50%, 69%,76%) and fluid administration in septic shock (50%, 42%, 75%) in Cohort A, Cohort B and Cohort C respectively.

## Conclusion

Baseline compliance with the SSC 3-hour bundle on level 1 wards was very low. An electronic sepsis order set was associated with marked improvement. Novel quality improvement methodology may be important to achieve optimal compliance with evidence-based guidelines and an electronic sepsis order set is recommended.
